# Neuroimaging studies of striatum in cognition part II: Parkinson's disease

**DOI:** 10.3389/fnsys.2015.00138

**Published:** 2015-10-08

**Authors:** Alexandru Hanganu, Jean-Sebastien Provost, Oury Monchi

**Affiliations:** ^1^Department of Clinical Neurosciences and Department of Radiology, Cumming School of Medicine, University of CalgaryCalgary, AB, Canada; ^2^Hotchkiss Brain Institute, University of CalgaryCalgary, AB, Canada; ^3^Centre de Recherche de l'Institut Universitaire de Gériatrie de Montréal, Université de MontréalMontréal, QC, Canada; ^4^Department of Psychology, Faculty of Arts and Sciences, University of MontrealMontreal, QC, Canada

**Keywords:** striatum, cognition, Parkinson's disease, neuroimaging, dopamine

## Abstract

In recent years a gradual shift in the definition of Parkinson's disease (PD) has been established, from a classical akinetic-rigid movement disorder to a multi-system neurodegenerative disease. While the pathophysiology of PD is complex and goes much beyond the nigro-striatal degeneration, the striatum has been shown to be responsible for many cognitive functions. Patients with PD develop impairments in multiple cognitive domains and the PD model is probably the most extensively studied regarding striatum dysfunction and its influence on cognition. Up to 40% of PD patients present cognitive impairment even in the early stages of disease development. Thus, understanding the key patterns of striatum and connecting regions' influence on cognition will help develop more specific approaches to alleviate cognitive impairment and slow down its decline. This review focuses on the contribution of neuroimaging studies in understanding how striatum impairment affects cognition in PD.

## Introduction

Parkinson's disease (PD) is a progressive neurodegenerative disorder that affects up to 2% of individuals aged 65 years and older (Rijk et al., 1997) and has an incidence of 14 per 100,000 individuals (Hirtz et al., 2007). In people over 70 years the incidence is much higher—160 per 100,000 individuals (Hirtz et al., 2007), and affects nearly 10% of people older than 80 years (von Campenhausen et al., 2005). Furthermore, the prevalence of PD is expected to double by 2030 (Dorsey et al., 2007). While PD is associated with a complex pathophysiology that can potentially affect most of the brain, the motor cardinal symptoms of PD are largely due to degeneration of dopamine (DA) neurons in the substantia nigra pars compacta (Albin et al., 1989). This pattern of neurodegeneration starts from dorsal striatum and extends to more ventral parts of the striatum as the disease progresses (Kish et al., 1988).

Since the second part of the 1980's it is becoming increasingly clear that cognitive deficits can be present even at the early stages of PD (Taylor et al., 1986; Taylor and Saint-Cyr, 1995; Dubois and Pillon, 1996). Initial investigations in mild to moderately affected PD patients emphasized deficits in executive functions (e.g., planning and set-shifting), which resemble those found in patients with frontal lobe damage (Taylor et al., 1986; Owen et al., 1990, 1992). These are consistent with the fronto-striatal dysfunction occurring in PD reported by our group and many others. However, non-frontal cognitive deficits including visuospatial and language function difficulties are also recognized in early-PD patients and at various stages of disease progression. In the context of this review we concentrate on the cognitive deficits that are most likely to originate from the fronto-striatal deficits. Indeed, striatal dysfunction in the context of cognitive deficits in PD is likely the most extensively studied amongst neurological and mental disorders affecting striatal functions.

Individuals who meet criteria for mild cognitive impairment (MCI) exhibit measurable cognitive deficits but those deficits are not severe enough to interfere significantly with daily life, nor reach criteria for dementia. MCI in PD patients can be identified using the MDS Task Force criteria (Litvan et al., 2012) and can be found in the early stages of the disease with up to 65% of patients at 1 standard deviation below normative values and in up to 42% of PD patients at 1.5 standard deviation (Aarsland and Kurz, 2010; Yarnall et al., 2014). Furthermore, PD patients with MCI have a higher risk of developing dementia compared with patients who do not have MCI (Emre et al., 2007; Kehagia et al., 2010).

Changes in dopaminergic availability are known to greatly affect fronto-striatal function in PD and to affect cognitive processes (Cools, 2006). Furthermore, dopaminergic therapy aimed at the motor symptoms of PD is likely to have an influence on cognition in PD. In this review we focus on how striatum and related dopamine dysfunction can affect cognition in PD and how dopaminergic medication can modulate those functions.

## Striatum organization

**Figure 1:**
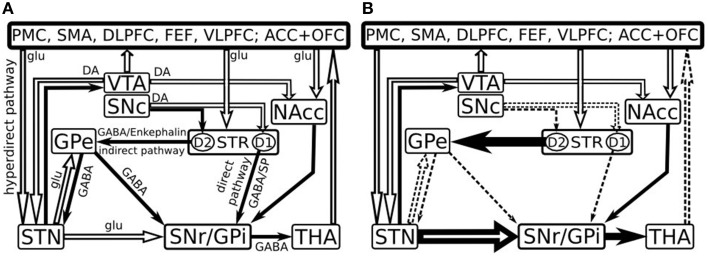
**Model of the basal ganglia connectivity in healthy (A) and in early Parkinson's disease (B) [Adapted after Kalivas and Nakamura (1999), Dudel et al. (2002), Nambu et al. (2002), Gubellini et al. (2009) and Cools (2006)]**. Open and filled arrows represent excitatory glutamatergic (glu) and inhibitory gabaergic (GABA) projections. PMC, Premotor cortex; SMA, supplementary motor area; DLPFC, dorsolateral prefrontal cortex; FEF, frontal eye fields; VLPFC, ventrolateral prefrontal cortex; ACC, anterior cingulate cortex; OFC, orbitofrontal cortex; VTA, ventral tegmental area; SNc, substantia nigra pars compacta; SNr, substantia nigra pars reticulata; NAcc, nucleus accumbens; GPe, external segment of the globus pallidus; GPi, internal segment of the globus pallidus; STR, striatum; STN, subthalamic nucleus; THA, thalamus; DA, dopamine; SP, Substance P; D1, D1 class DA receptors; D2, D2 class DA receptors.

In humans, striatum is attributed to a complex consisting of the caudate nucleus and putamen (dorsal striatum) as well as the most ventral part of the caudate nucleus, the ventral part of the putamen and the nucleus accumbens (ventral striatum) (Grahn et al., 2009). The striatum connections are organized into direct and indirect pathways which are based on the striatal output projections (Figure 1) (Albin et al., 1989). The nigral neurons project via the nigrostriatal pathway to the striatum (Samii et al., 2004) and provide dopamine (DA), which reinforces cortically initiated activation of a particular basal ganglia-thalamo-cortical circuit. This is achieved by facilitating conduction through the circuit's direct pathway, which has a net excitatory effect on the thalamus, and suppressing conduction through the indirect pathway, which has a net inhibitory effect on the thalamus (Alexander and Crutcher, 1990).

The direct pathway consists of neurons that predominantly express D1 DA receptors, substance P and dynorphin (Haber and Nauta, 1983; Gerfen et al., 1990; Steiner and Gerfen, 1999). They project from the striatum to the internal segment of the globus pallidus (GPi) and to substantia nigra pars reticulata (SNr), eliciting a phasic inhibition of the GPi/SNr and promoting desired movements (Alexander et al., 1986). The indirect pathway on the other hand, is comprised of striatal neurons that express predominantly D2 receptors (Gerfen et al., 1990), met-enkephalin and neurotensin (Haber and Nauta, 1983; Steiner and Gerfen, 1999). These neurons project to external portion of the globus pallidus (GPe) and the subthalamic nucleus (STN). Since GPe tonically inhibits the SNr, activation of the indirect pathway performs an inhibition of the GPe and a disinhibition of the SNr resulting in increased inhibitory activity of the SNr over the thalamus and thus exerting an inhibition or termination of the movement (Alexander et al., 1986). DA is a modulatory neurotransmitter and mediates synaptic plasticity both morphologically (Li et al., 2004) and electrophysiologically (Calabresi et al., 1992; Reynolds et al., 2001). The phasic activation of DA-ergic neurons exert a net excitatory effect on the direct pathway and an inhibitory effect on the indirect pathway (Delong and Wichmann, 2007), thus facilitating or inhibiting the functions that depend on striatum activity—movements, learning, working memory, attention, and other. The hyperdirect pathway originates from the axon collaterals of pyramidal tract neurons and project to the STN, thus it receives and displays activity directly related to movement (Giuffrida et al., 1985). This path is faster than the direct and indirect pathways and it exerts a powerful excitatory effects on the STN further activating GPi neurons and resulting in the inhibition of large areas of the thalamus and cortex that are related to the selected movements and or competing programs (Nambu et al., 2002).

Striatum connectivity with the cortex has been initially studied in monkeys and later on using magnetic resonance diffusion tensor imaging in humans, *in vivo* (Lehéricy et al., 2004). The head of the caudate and the rostral putamen were shown to be connected primarily to the frontal lobe (medial, ventral, dorsolateral prefrontal cortex (PFC), frontal pole, pre-supplementary motor area); the posterior putamen revealed anatomical connectivity with posterior supplementary motor area, motor and sensory areas, while the ventral striatum was connected to orbitomedial frontal cortex, temporal pole, amygdala, and hippocampus (Lehéricy et al., 2004). This connectivity pattern is in line with previously described frontostriatal circuits which were segregated into “motor” (supplementary motor area → putamen), “limbic” (anterior cingulate → nucleus accumbens), and “associative” domains, specifically the oculomotor loop (frontal eye fields → caudate nucleus), the dorsolateral circuit (dorsolateral prefrontal cortex → dorsolateral caudate nucleus), and ventral orbital loop (orbitofrontal cortex → ventromedial caudate nucleus) (Alexander et al., 1986, 1989).

In PD, the loss of DA-ergic inputs from substantia nigra pars compacta (SNc) leads to an enhanced cholinergic signaling that in turn produces disinhibition of D2-receptor-containing striatum neurons. Both the glutamatergic inputs from the cortex to the striatum and from the STN to the output nuclei are significantly enhanced while the GABA-ergic inputs from the striatum and the external segment of the pallidum to the output nuclei become impaired. This imbalance leads to an increased inhibitory GABA-ergic output to the thalamus and to decreased glutamatergic thalamocortical feedback, which results in hypokinetic motor symptoms as well as hypoactivity in the cognitive functions, as it has been suggested previously (e.g., Owen, 2004). Specifically the impairment is attributed to those cognitive functions that involve the striatum—memory, visuospatial function, attention and working memory, executive decision, language, emotions. Previous pathological (Rinne et al., 1989; Paulus and Jellinger, 1991) and positron emission tomography studies confirmed a correlation between caudate DA loss and neuropsychological performance in PD patients (Marié et al., 1999; Brück et al., 2001) suggesting a preferential role for this system in cognitive impairment (Ito et al., 2002).

In patients with PD, cognitive impairment is frequently observed, especially with respect to executive functions (Cools, 2006), which are cognitive mechanisms through which performance is optimized in situations requiring the simultaneous operation of different processes (Morris et al., 1990). Executive control is especially active when the action or response is novel or complex (Norman and Shallice, 1986) such as pursuing a long-term goal requiring the completion of multiple intermediate sub-goals and behavior. Executive functioning refers to (1) switching attention between different processes (attentional set-shifting); (2) directing attention to a relevant stimulus and inhibiting the irrelevant stimuli (goal-directed action); (3) coding and checking the contents of memory storage (working memory) and (4) concept formation and planning strategies (Cools, 2006).

## Striatal impairment in attentional set-shifting

Attentional set-shifting is probably the most widely studied executive deficit in PD (Cools et al., 1984; Downes et al., 1989; Owen et al., 1992; Van Spaendonck et al., 1996; Monchi et al., 2004) but it is also impaired in patients with prefrontal cortex (PFC) lesions (Gotham et al., 1988; Cools et al., 2001b). The Wisconsin Card Sorting Task (WCST) is the most commonly used test to assess set-shifting in humans (Milner, 1963; Nelson, 1976) and involves matching test cards to one of four reference cards according to three possible classification rules. Participants must use feedback in order to select the correct rule, as it is not explicitly given. After a fixed number of correct matches, the rule is changed without notice and participants must switch to a new rule for classification, constituting a set-shift. Using an event-related fMRI protocol in healthy controls, we showed the implication of two cortico-striatal loops during different WCST stages. When planning a set-shift, the ventrolateral PFC (VLPFC), the caudate nucleus, and the thalamus were significantly activated, while the execution of the set-shift solicited the posterior PFC and the putamen (Monchi et al., 2001). The same protocol was subsequently used in early-stage PD patients (following overnight DA-ergic medication removal) and matched controls (Monchi et al., 2004). The results reduced activation in the PD group in the VLPFC when receiving negative feedback and the posterior prefrontal cortex when matching following negative feedback. Activity in these areas specifically correlated with the striatum in controls. By contrast, greater activation was found in the PD group in areas that were not co-activated with the striatum in controls. These results suggest that both nigrostriatal dopamine depletion and intra-cortical dopamine deficiency may play a role in cognitive deficits in PD, depending on the involvement of the striatum in the task at hand (see below). Furthermore, using the same protocol in early PD patients divided into two groups based on the presence of MCI, PD patients without mild cognitive impairment (PD-non-MCI) “off” medication, revealed patterns of activation similar to healthy individuals from our previous studies when planning a set-shift (Monchi et al., 2004, 2007; Jubault et al., 2009) with significant activation in the VLPFC and caudate nucleus. In contrast, PD patients who had mild cognitive impairment (PD-MCI) had no significant activation in these regions (Nagano-Saito et al., 2014). Also, when matching following the set-shift, the PD-non-MCI group revealed significant activity in the premotor cortex but not the putamen resembling previous PD “ON” medication results (Jubault et al., 2009). Similar results have been observed in PD-MCI and PD-non-MCI patients while performing a working memory task (Ekman et al., 2012; Monchi and Stoessl, 2012). The finding of striatal activation in the WCST is consistent with the possibility that the basal ganglia are involved in selecting the relevant action among competing motor responses (Mink and Thach, 1993).

## Striatal impairment in goal-directed action and planning strategies

The Tower of London (Shallice, 1982) has often been used to analyze the failure of goal-directed action and planning strategies in PD. During this test patients are required to move a set of three colored balls around in “pockets” or “socks” to match a goal arrangement presented at the top of the screen (Owen et al., 1990). This task involves several stages (1) evaluation of the overall situation—understanding the differences between the initial state and the goal; (2) defining the sequence of moves that are necessary to achieve the goal; and (3) executing the correct solution. This task has been shown to recruit the caudate nucleus and the dorsolateral prefrontal cortex (Owen et al., 1996). In healthy controls, when the difficulty of the problem was increased, there was an increase in caudate nucleus activity (Owen et al., 1996; Dagher et al., 1999), while increasing the number of moves resulted in increased activity in the putamen (Dagher et al., 1999). PD patients at the early stage of the disease spent more time on planning the strategy than the controls (Owen et al., 1992). Furthermore, according to a functional neuroimaging study, the right caudate nucleus activity has been reported to be impaired in patients with mild PD compared with healthy controls when performing the task (Dagher et al., 2001). Interestingly, an eye-gaze behavior study has shown that PD patients seemed to fail to attend appropriately to the goals of the task (Hodgson et al., 2002).

It is clear that striatum impairment in PD leads to impaired goal-directed action and planning strategies. However, there may be two different reasons for this: (1) the inability to identify and maintain relevant goal information, or (2) bradyphrenia, i.e., slower thinking and an increase in the time taken to solve problems while the process of problem solving is preserved. Additionally, problem solving is likely not uniquely a striatal function but also a frontal one. Previous studies reported that impaired problem solving was present in patients with frontal lobe lesions (Owen et al., 1990). Indeed, it has been reported that abnormal striatal activation in PD was accompanied by a performance deficit similar to the one observed in patients with frontal lobe damage (Owen et al., 1993), but there were no abnormalities in the regional cerebral blood flow in the prefrontal cortex (Dagher et al., 2001). Additionally, patients with frontal lobe damage did not reveal longer thinking times during Tower of London task but they required more moves to reach the goal (Owen et al., 1990). This would suggest that striatum is responsible for the goal-directed deficit. Furthermore, according to some reports, medicated PD patients were shown to be impaired in the amount of time spent thinking about the solution (planning) (Owen et al., 1992), while another Tower of London study reported that even if PD patients directed their eyes toward the workspace, during planning like healthy controls, they divided their attention equally between the goals and the workspace. This suggests that the planning deficit in PD is due to abnormal encoding and maintenance of current goals (Hodgson et al., 2002). In conclusion to this section, it seems that the striatum is responsible for efficient goal-directed actions (encoding and maintaining the goals), but that the striatal dysfunction and the increased inhibitory GABA-ergic output to the thalamus that is present in PD, lead to both to an increased amount of time spent thinking and planning as well as with respect to goal-directed actions.

## Striatal impairment in working memory and decision-making

Dopamine (DA) innervation to the prefrontal cortex and the striatum is critical for normal decision-making and working memory function both at the cellular and behavioral levels (Goldman-Rakic, 1995; Williams and Goldman-Rakic, 1995). Working memory is important for the ability to hold and manipulate information when involved in problem solving and decision making (Frank et al., 2007). Specifically, D1-receptor activation in the striatum (the direct pathway) has been shown to stabilize neuronal ensembles with high activity in the PFC, and this was suggested to be a method of maintaining the information within working memory (Durstewitz and Seamans, 2006). On the other hand, D2-receptors (the indirect pathway) appears to destabilize neuronal ensembles and makes them more susceptible to neuronal input, a state that has been considered as updating the information in working memory (Durstewitz et al., 2000). Working memory performance is also compromised in both PD patients and those with frontal lobe lesions, even if the reasons of impairment are different. One study showed that both groups of patients are impaired on a test that requires the selection and sequencing between a series of sub-goals (Owen et al., 1990, 1992). Participants were required to search for hidden “tokens” through boxes and to avoid the boxes that previously were associated with reward (between search) and to avoid returning to boxes that were previously opened and shown to be empty (within search) (Owen et al., 1990). Both groups of patients—PD and frontal lobe-lesioned—showed “between search” errors when compared with healthy controls (Owen et al., 1992), but PD patients did not have significant “within search errors,” unlike frontal lobe-lesioned ones (Owen et al., 1996). Other studies also confirmed that working memory dysfunctions was associated with reduced activity (Lewis et al., 2003) and abnormal blood flow (Owen et al., 1998) in the caudate nucleus in PD patients who were executively impaired. Additionally, structural studies revealed that larger caudate size in the individuals deemed at ultra-high risk of psychosis was associated with greater errors on a spatial working memory task (Hannan et al., 2010). These results underline that frontal lobe is associated with performing the search and adopting strategies and organization with working memory (because the frontal lobe is likely less impaired in early PD) while the caudate nucleus becomes involved when there is a necessity of self-generated novel actions that would alternate effectively between important sub-goals and consequently modifies the behavior.

## Striatal impairment in procedural learning

Learning associations between stimuli and responses or categories is an important ability across species (Wise and Murray, 2000), and the striatum, particularly the caudate nucleus, plays a key role in such learning (Seger and Cincotta, 2005). The cortico-striatal circuitry has been emphasized to have a critical role in learning, and specifically in supporting the “procedural” learning system (Eichenbaum and Cohen, 2001; Shohamy et al., 2004). In fact, studies revealed that PD patients were slower during learning of an associative task, but they were unimpaired in the process of transferring the information, while patients with hippocampal atrophy showed the opposite pattern—good initial learning and impaired ability to transfer when familiar stimuli were presented in novel recombination (Swainson et al., 2000; Myers et al., 2003). Further investigations using a behavioral task in which participants viewed arbitrary visual patterns and used them to predict one of two possible outcomes, revealed that caudate nucleus contributes to learning in two distinct ways. Activity associated with successful classification learning (correct categorization) is concentrated to the body and tail of the caudate nucleus, while activity associated with feedback processing (the result of incorrect categorization) is concentrated to the head of the caudate nucleus (Seger and Cincotta, 2005). Patients with early PD were shown to be much more impaired at rule-based category learning than at information classification learning (Ashby et al., 2003). This is in line with the progressive neurodegeneration pattern in PD (Kish et al., 1988) with an increased rate in the head of the caudate nucleus—hence worse functioning, and a lower extent of neurodegeneration in the caudate nucleus tail.

## Interrelation between striatum and prefrontal cortex

Executive functions are widely associated with the frontal lobe, in particular with the dorsolateral prefrontal cortex (DLPFC), which is involved in certain aspects of working memory (Petrides, 2000) and cognitive flexibility (Milner, 1963; Goldman-Rakic, 1987). Specifically, anatomical studies showed that the most rostrodorsal extent of the caudate head is connected with the DLPFC (Yeterian and Pandya, 1991) while PET studies demonstrated increased PFC activation in PD patients performing tests of executive function (Owen et al., 1998; Dagher et al., 2001). From this point of view, in PD patients one would expect diminished activity in the DLPFC as well, considering that within the caudate nucleus DA depletion is greatest in the caudate head (to a maximum of about 90%). Nevertheless, our previous studies showed that increase and decrease in PFC activity in patients with PD is related to whether the striatum is necessary for the task or not (Monchi et al., 2004, 2007, 2010). Specifically, we revealed a decrease in PFC activity (hypoactivation) of patients with PD off medication compared with healthy controls, for tasks that recruit the striatum in healthy controls (i.e., planning a set-shift). In contrast, when performing tasks that do not require the striatum (i.e., task execution without changing the rule) in healthy controls, patients with PD showed significant prefrontal and parietal increases in activity—hyperactivation—usually unrelated to the task (Monchi et al., 2007). Previous investigations in PD patients “on” medication compared to those in the “off” state suggested that DA level accounted for the greater PFC activation in PD “on” medication (Cools et al., 2002; Mattay et al., 2002). These hyperactivations can be explained by an increase of DA support through the mesocortical projections, by which neurons from the ventral tegmental area (VTA) and the medial SNc project to the frontal lobe (Tzschentke, 2001; Grahn et al., 2009). Unlike the nigrostriatal DA-ergic system, which refers to the SNc DA [and which has been termed also as mesostriatal, because it refers to the SNc-VTA complex (Lindvall et al., 1977)], the VTA DA-ergic neurons project to the PFC (mesocortical pathway) and to the ventral striatum (mesolimbic pathway) (Björklund and Dunnett, 2007). In fact, it has been shown that cortical DA has a critical role in executive functions and high-level cognition (Murphy et al., 1996; Watanabe et al., 1997). Since DA neurons in the substantia nigra degenerate much earlier in the disease than neurons in the VTA (Agid et al., 1987a,b; Kish et al., 1988), activity during cognitive tasks of the cortical regions could be modulated by DA replacement through the mesocortical pathway (Tzschentke, 2001; Grahn et al., 2009).

On the other hand, our studies revealed that levodopa showed no effect on the activity of the cognitive fronto-striatal loop which included the DLPFC and the caudate nucleus, despite a significant effect on the activity of motor regions (Jubault et al., 2009; Martinu et al., 2012). A possible explanation for this increased activity in the DLPFC might be that mesocortical projections innervate predominantly the medial PFC, the infralimbic and prelimbic subareas (Tzschentke, 2001), hence PFC reveals a compensatory pattern not associated with DA concentration. This would suggest that even if in healthy controls the frontal cortex might not normally get involved in certain cognitive functions of the striatum, it becomes engaged in order to maintain a specific activity, as suggested previously (Samuel et al., 1997). Another explanation is that mesocortical projections have a diminished responsiveness to DA agonists and antagonists (Bannon and Roth, 1983), hence the absence of any effect on the activity of the cognitive fronto-striatal loop in our study. Nevertheless, a recent fMRI study in de-novo (untreated) PD patients during a set-shifting task, indicated that some of these frontal and parietal hyper-activation may be compensatory (Gerrits et al., 2015). It is likely that these hyperactivity patterns can represent both a mesocortical imbalance and compensation, depending on various factors, including the exact nature of the task, the advancement of the disease and the amount and type of dopamine medication being taken.

## Ventral striatum impairment and cognitive changes

Ventral striatum includes nucleus accumbens, rostral/ventral caudate nucleus and putamen. These regions are connected with orbital, medial (Haber et al., 1995) and ventral PFC (Yeterian and Pandya, 1991) forming the limbic loop, and are involved in emotional processing, motivational and stereotyped behavior, attention deficit disorder, hyperactivity disorder, compulsive disorders, Tourette's syndrome (Grabli et al., 2004), and reversal of stimulus reward associations (Nauta, 1971; Rolls, 2000). Furthermore, the nucleus accumbens is essential in integrating cortical and limbic information into goal-directed behavior (Pennartz et al., 1994). Several studies reported that in the earlier stages of PD development, DA depletion is restricted to the putamen and the dorsal caudate nucleus, while in the later stages DA depletion progresses to the more ventral parts of the striatum and the mesocortico-limbic DA-ergic system (Rosvold, 1972; Kish et al., 1988; Swainson et al., 2000; Cools et al., 2001a). This uneven pattern of striatal DA loss has been confirmed by postmortem neurochemical analysis in patients with PD (Kish et al., 1988). The different spatiotemporal progression of DA depletion within the striatum and the terminal distribution of its cortical afferents may be the best explanation for the evolving pattern of cognitive impairments observed in PD patients (Gotham et al., 1988). Specifically, we previously showed that PD patients who had MCI presented a higher rate of volume diminishment over time in the nucleus accumbens and a higher rate of cortical thinning in comparison to PD patients without MCI and to healthy controls (Hanganu et al., 2014).

On the other hand, the different level of DA depletion leaves room for DA overdosage due to DA-ergic treatment. The “over-dose” hypothesis has been discussed by many studies previously (Gotham et al., 1988; Swainson et al., 2000; Cools et al., 2001a) and it states that levodopa doses necessary to remedy the DA lack in severely depleted brain areas, such as the putamen, would detrimentally “over-dose” relatively intact brain areas in early PD, such as the ventral striatum and its connections to the orbital PFC. In fact, previous studies reported the impairment of ventral striatum in early disease with respect to DA therapy. Learning was most commonly impaired in PD patients tested on DA replacement therapy. Cognitive tasks such as probabilistic reversal learning, that challenges the ventral frontostriatal circuit (ventral PFC and ventral striatum), revealed decreased performance in PD patients with DA-ergic treatment, although PD patients off medication showed similar performance to controls (Cools et al., 2001a; Torta et al., 2009; Jahanshahi et al., 2010). Other studies in PD reported impaired learning of discrimination tasks (Shohamy et al., 2006) and sequences associated with DA-ergic medication usage (Feigin et al., 2003; Seo et al., 2010; Tremblay et al., 2010). DA treatment in PD patients also yielded reduced facilitation for consecutive, consistent stimulus–stimulus pairings in a selection task compared to normal implicit learning (MacDonald et al., 2011). Furthermore, once a stimulus-reward association was learned, reversing probabilities of stimulus-reward associations was also impaired in PD patients on medication (Swainson et al., 2000; Cools et al., 2001a, 2006; Graef et al., 2010; MacDonald et al., 2011). Time estimation was also affected. Increased response time was reported in a simple reaction task in PD patients after administration of DA in comparison to PD patients off medication and healthy controls (Müller et al., 2001).

The nucleus accumbens is also thought to be involved in inhibitory control processes (Christakou et al., 2004) and increased impulsive choice (Cardinal et al., 2001). In the case of PD patients, impulsive betting was noted after DA treatment, despite appropriate and deliberate decision making (Cools et al., 2003; Torta et al., 2009). Other studies also reported an increased number of impulse control disorders in PD patients on medication, such as pathological gambling, compulsive sexual behavior, compulsive buying, and binge eating (Ray and Strafella, 2010; Weintraub et al., 2010).

In line with the “overdose” hypothesis, studies reported an improvement of functions associated with ventral striatum during “off” state. Specifically, improvements were shown in the learning to avoid choices that lead to negative outcomes in comparison to learning from positive outcomes (Frank et al., 2004). Other studies reported improvement in associative conditional learning (Gotham et al., 1988) and probabilistic reversal learning (Swainson et al., 2000; Cools et al., 2001a). These functions have been associated with the ventral striatum and the ventral PFC including the orbitofrontal cortex (Dias et al., 1996; Passingham et al., 2000; Cools et al., 2001a). On the other hand, levodopa withdrawal has been shown to impair PD patients in verbal fluency (Gotham et al., 1988), set-shifting tasks (Cools et al., 2001a), spatial recognition memory task (Swainson et al., 2000), and in tasks requiring trial and error (Frank et al., 2004), functions that are associated especially with the dorsal caudate and DLPFC (Owen et al., 1990).

The nucleus accumbens has also been heavily associated with reward processing (Schultz et al., 2000). Indeed various studies have investigated the effect of dopamine replacement therapy in PD in the context of stimulus-reward learning. It was reported that once a stimulus-reward association was learned, reversing probabilities of stimulus-reward associations was impaired in PD patients on dopaminergic medication (Swainson et al., 2000; Cools et al., 2001a, 2006; Graef et al., 2010; MacDonald et al., 2011). A two-fold study by MacDonald et al. (2011) used a simple selection task to elucidate functions mediated by the ventral and dorsal striatum respectively with fMRI in healthy individuals, and to better understand the cognitive effects of dopamine replacement in PD testing them behaviourally with the task once ON their usual dose of DA-ergic medication and once following overnight withdrawal. In healthy individuals, the congruent condition that involved consistent stimulus–stimulus associations across two consecutive events, without reward or feedback being provided, solicited significant ventral striatum activation, while the incongruent condition that involved conflicting relationships across two consecutive events (baring similarity to set-shifting) solicited significant activation in the head of the caudate nucleus. In PD patients DA replacement impaired encoding and facilitation of consistent stimulus–stimulus relations across trials in the congruent condition relying on ventral striatum, while it enhanced interference related to assimilating conflicting influences on selection across trials in the incongruent condition relying on dorsal striatum. These studies gave support that impairments specific to the ventral striatum in PD patients can be explained by the “over-dose” hypothesis (Gotham et al., 1988; Swainson et al., 2000; Cools et al., 2001a).

## Striatum and cerebellum relationship in PD with respect to cognitive changes

It has been suggested that cerebellum may compensate for impaired basal ganglia cognitive function (Strick et al., 2009; Appel-Cresswell et al., 2010) and several fMRI studies reported evidence that cerebello-thalamo-cortical loop increases its activity to compensate for degeneration in the striato-thalamo-cortical loop to maintain a near-normal motor function (Glickstein and Stein, 1991). This compensational patterns are further explained by the presence of two main cerebellum circuits—the “motor” loop, projecting from the motor and premotor cortex to the dentate nucleus, and the “prefrontal” loop, that connects the posterior PFC (Brodmann Area 9/46) and the dentate nucleus (Kelly and Strick, 2003). The prefrontal loop has been associated with cognitive functions (Strick et al., 2009). Furthermore, recent evidence reported direct connections between striatum and cerebellar circuits (Bostan and Strick, 2010)—a tri-synaptic connection between the GPe and the dentate nucleus (Hoshi et al., 2005) and a bi-synaptic projection from the STN to the cerebellar cortex via pontine nuclei (Bostan et al., 2010). Altogether, this gives strong support that cerebellum is an organized compartment used for the integration of non-motor functions such as emotion, working memory, and language, as suggested previously (Stoodley and Schmahmann, 2009).

In PD patients, it has been argued that cerebellum has a compensatory role because when patients were off medication they revealed increased activation in the cerebellum during externally guided motor tasks, compared with healthy controls and with PD patients on medication (Rascol et al., 1997; Cerasa et al., 2006; Yu et al., 2007), as well as during internally generated movements (Cerasa et al., 2006; Yu et al., 2007). Furthermore, a negative correlation has been reported between cerebellum and contralateral putamen in PD patients off medication, suggesting that cerebellum activity increases in order to compensate the reduced putamen activation (Yu et al., 2007). Furthermore, PD patients off medication, when compared to healthy controls, revealed decreased functional connectivity in the supplementary motor area, DLPFC and putamen, while cerebellum, primary motor cortex, and parietal cortex showed increased connectivity (Ng et al., 2010). Levodopa administration relatively normalized this connectivity pattern in PD patients.

It is also worth to note that one of cerebellum functions regarding cognition, is its timing capacity. Patients with cerebellar damage have difficulties accurately producing and perceiving time intervals (Ivry and Keele, 1989) and due to this, internal cognitive states may no longer be appropriately selected and sequenced at a fine level, which may exhibit problems with task-shifting and other forms of executive control (Strick et al., 2009). Our previous work with set-shifting tasks reported a decrease in timing activity in the prefrontal regions of patients with PD off medication compared to healthy controls for tasks that require the striatum in healthy controls (Monchi et al., 2007).

On the other hand, several researchers presented contrasting results. Hosokai and colleagues didn't find any significant increase in cerebellar metabolism both in PD-demented and PD-MCI patients in comparison to PD patients without MCI (Hosokai et al., 2009). Furthermore, one previous PET study reported a negative correlation between the cerebellum metabolism and regional cerebral blood flow both in PD patients and in healthy controls during procedural memory processes (Dagher et al., 2001). These results indicate that the cerebellum does not necessarily compensate for cognitive impairment of basal ganglia origin. Habas et al. (2009) also questioned cerebellum's cognitive functions due to failure of some studies to find significant cognitive impairment in cerebellar lesion patients (Helmuth et al., 1997; Thier et al., 1999; Haarmeier and Thier, 2007). Furthermore, in their reports Glickstein (2006) and Glickstein et al. (2011) failed to replicate previous studies reporting cerebellar contribution to cognition in PD even when using a low threshold. Finally, it has also been argued that the cerebellum activation in cognition can be subject to contamination by skeletal or eye movements (Strick et al., 2009).

In summary, the hypothesis that cerebellar circuits may compensate for impaired basal ganglia cognitive function in PD, as suggested previously (Stoodley and Schmahmann, 2009; Appel-Cresswell et al., 2010), still remains unresolved and further investigations are necessary.

## Conclusion

Striatum impairment in PD is caused initially by a diminished modulatory effect of DA from the SNc which results in enhanced GPi activation, increased inhibitory output to the thalamus and decreased thalamocortical feedback. Such a dysregulation destabilizes neuronal input, affecting the fronto-striatal loops, and impairing cognitive function. DA replacement therapy increases the striatum modulatory function, yet it also induces an overdose effect on the structures that have a relatively normal DA level, impairing their functions (e.g., ventral striatum) and as the disease continues to progress, cognitive impairment progresses along. In the initial stages of PD development PFC maintains a normal cognitive activity, either due to mesocortical DA sources or due to compensational patterns, which makes the cognitive impairment profile to be restricted to the dorsal striatum dysfunction. Nevertheless, the present DA replacement therapies cannot avoid further cognitive decline, since it has been shown that up to 75% of PD patients eventually develop dementia (Aarsland and Kurz, 2010). Furthermore, some studies reported that intellectual deterioration does not seem to result from dysfunction of DA-ergic mechanisms (Pillon et al., 1989). Taken together, these data suggest that cognitive disturbances in PD are related both to nigrostriatal, mesolimbic, and mesocortical DA-ergic systems as well as non-striatal and non-dopaminergic origins. Specifically, abnormalities in other systems have been found in PD (Agid et al., 1987a)—the cholinergic septo-hippocampal and innomasto-cortical pathways, the noradrenergic coeruleo-cortical neurons and the serotoninergic neurons (dorsal raphe nuclei) (Ruberg and Agid, 1988; Jellinger, 1999). Relationships between lesions of cholinergic (Sadeh et al., 1982; Bohnen et al., 2006; Ziabreva et al., 2006) and noradrenergic (Cash et al., 1987; Delaville et al., 2011; McMillan et al., 2011) systems and cognitive impairment in PD patients have been reported previously. Furthermore, degeneration of cortical neurons or decreases in cortical peptide concentrations (Agid et al., 1987b) may also contribute to cognitive impairment in PD. These systems should also be addressed in order to encircle the whole extent of striatal and non-striatal cognitive dysfunction in PD.

### Conflict of interest statement

The authors declare that the research was conducted in the absence of any commercial or financial relationships that could be construed as a potential conflict of interest.
